# Prostatic Artery Embolization for the Treatment of Benign Prostatic Hyperplasia: A Retrospective Single-Center Study

**DOI:** 10.7759/cureus.73064

**Published:** 2024-11-05

**Authors:** Rupam Deori, Diwakar Neelakandan, Shivakumar M Algud, Renu Thomas, Manish K Yadav, Ajit N Vaidya

**Affiliations:** 1 Urology, Kerala Institute of Medical Sciences (KIMSHEALTH), Thiruvananthapuram, IND; 2 Interventional Radiology, Kerala Institute of Medical Sciences (KIMSHEALTH), Thiruvananthapuram, IND

**Keywords:** benign prostatic hyperplasia, international prostate symptom score, lower urinary tract symptoms, prostatic artery embolization, quality of life

## Abstract

Introduction

The purpose of this study is to assess the effectiveness and short-term outcomes of prostatic artery embolization (PAE) in Indian patients suffering from benign prostatic hyperplasia (BPH).

Methods

This retrospective analysis was performed at a single center and included 25 patients with BPH who received PAE from January 2019 to June 2023. The symptoms of patients had been assessed utilizing the International Prostate Symptom Score (IPSS) and the Quality of Life (QoL) questionnaire. The prostate volume and post-void residual (PVR) volume were assessed using transabdominal ultrasonography prior to and following the embolization. The procedure occurred in the interventional radiology suite with the patient under conscious anesthesia, employing polyvinyl alcohol (PVA) particles. Prostate volume, PVR, IPSS, and QoL scores had been assessed at a follow-up three months later.

Results

Twenty-five males, with an average age of 76.56 years, underwent PAE. Bilateral embolization was conducted in 23 patients, whereas unilateral embolization was executed in two cases. Following a duration of three months, the average maximum improvement was as defined: IPSS, 10.44 ± 2.91; QoL score, 1.80 ± 0.81; prostatic volume decrease, 49.40 ± 24.13 cc (43% ± 13.95); and PVR volume, 70.08 ± 39.85 mL (52% ± 14.14) (p < 0.001 for all) as evaluated by the paired t-test.

Conclusion

PAE is a secure and efficacious intervention for BPH, yielding favorable short-term outcomes for lower urinary tract symptoms.

## Introduction

Lower urinary tract symptoms (LUTS) in older men are frequently caused by benign prostatic hyperplasia (BPH). It is defined as the nonmalignant development of prostate tissue. This illness typically becomes more common as people age. Autopsy studies reveal that BPH affects 50% to 60% of men in their 60s and 80% to 90% of those over 70 years old [1]. First-line therapies are usually conservative measures like medication and lifestyle modifications. Other therapy alternatives are explored for patients who do not respond well or who exhibit symptoms of hematuria, urine retention, recurrent urinary tract infections (UTIs), or renal insufficiency. Among these alternatives are holmium laser enucleation of the prostate (HoLEP), prostatic urethral lift (PUL), transurethral vaporization of the prostate (TUVP), and transurethral resection of the prostate (TURP) [2].

Between medicinal care and surgical alternatives for BPH, prostatic artery embolization (PAE) has recently become a viable alternative treatment for LUTS. In the United Kingdom, the National Institute for Health and Care Excellence (NICE) arrived at the conclusion in 2018 that PAE was sufficiently safe and effective to recommend it for carefully selected patients [3]. The absence of general anesthesia, a good safety record for high-risk patients, the ability to continue taking antiplatelet drugs, a decreased risk of surgery, and a decreased chance of sexual health side effects like erectile dysfunction or retrograde ejaculation are some advantages of PAE [4,5]. In 2000, DeMeritt et al. performed the first prostate embolization to treat post-biopsy hematuria in a BPH case, introducing PAE [6]. The first PAE for BPH was carried out in 2010 by Carnevale et al., who promoted it further by demonstrating symptomatic relief and a decrease in prostate volume in two patients [7]. This study aimed to assess the effectiveness and short-term outcomes of PAE for the treatment of symptomatic BPH.

## Materials and methods

Study participants and procedural management

The medical records of 25 patients with bothersome LUTS, admitted to the Kerala Institute of Medical Sciences in Thiruvananthapuram, India, were retrospectively reviewed. Patients with BPH who had PAE between January 2019 and June 2023 were the subject of this study. Prior to this, all participants had not responded to medical treatment and had been counseled to consider surgery. Additionally, the sample comprised self-referred patients seeking therapy in the interventional radiology department, high-risk surgical patients, and patients who preferred non-surgical options. The International Prostate Symptom Score (IPSS) and the Quality of Life (QOL) Questionnaires were used to measure symptoms. Transabdominal ultrasonography was used to measure the prostate volume as well as post-void residual (PVR) volume prior to and following embolization. Before the procedure, all patients' serum prostate-specific antigen (PSA) levels were measured. Using polyvinyl alcohol (PVA) particles, PAE was carried out in the interventional radiology suite while the patient was under conscious sedation. Each patient gave their informed written consent before the procedure.

Technique

Every patient received a detailed explanation of the embolization procedure. Prostate volume and PVR were measured using abdominal pre-embolization ultrasonography. One day before the procedure, the patients were admitted. In a biplane cath lab, prostate embolization was performed using either the Philips FD 20 monoplane system (Philips Healthcare, Andover, MA, USA) or the Philips Azurion 7 biplane system (Philips Healthcare, Andover, MA, USA). The bladder was catheterized and clamped to enhance the visualization of the vascular anatomy. A solution of 50% contrast mixed with saline was used to fill the Foley's bulb, improving its visibility in the cath lab and providing an estimate of the prostate's location. Antibiotics were initiated upon admission and continued for seven days after the procedure. Pre-procedure urine cultures were performed to inform the antimicrobial therapy.

**Figure 1:**
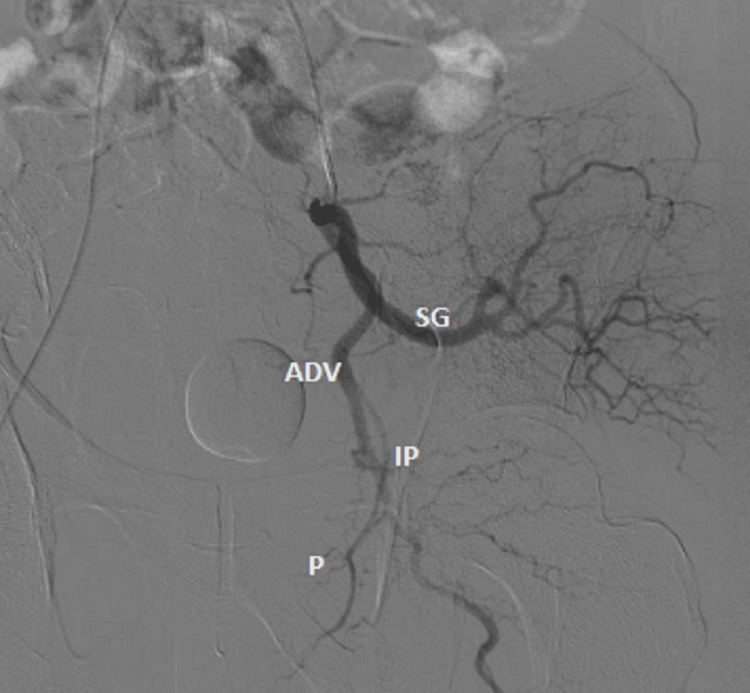
Internal iliac artery angiogram showing Yamaki A branching pattern of gluteo-pudendal trunk and origin of prostatic artery ADV, anterior division of internal iliac artery; IP, internal pudendal artery; P, prostate artery; SG, superior gluteal artery

PAE was conducted using local anesthesia. Access to the right femoral artery was achieved with ultrasound guidance and a 4 Fr Bernstein catheter (Cordis, Bloomington, IN). In cooperative and suitable patients, the radial artery approach was also utilized. An angiographic assessment of the aorta and selective angiography of the internal iliac artery were performed in order to investigate the anatomy of the prostate artery (Figure 1).

**Figure 2:**
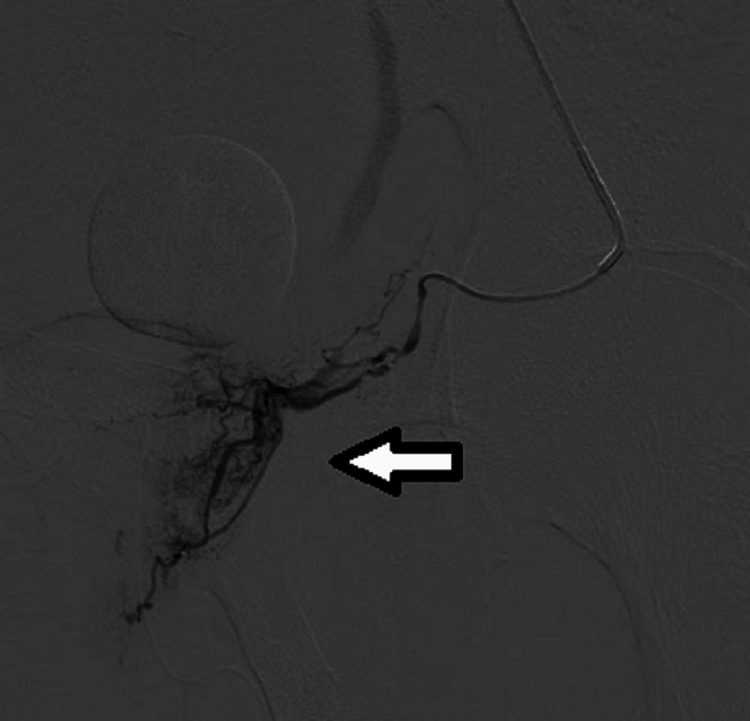
Selective intubation of prostatic artery showing cork screw feeders supplying the central gland with raised parenchymal blush

After deciphering the arterial anatomy, super-selective catheterization was conducted using a 1.9F Masters Parkway Soft microcatheter (Asahi Intecc Co., Ltd., Aichi, Japan). The position was verified with a follow-up angiographic run (Figure 2).

**Figure 3:**
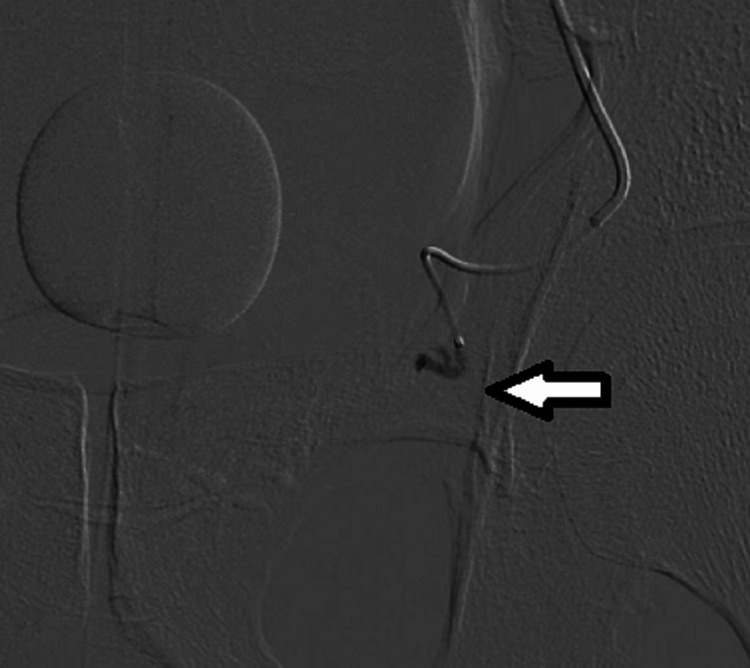
Post-embolization image showing complete stasis of contrast without any parenchymal blush

Embolization was conducted by utilizing non-spherical PVA particles measuring 150-250 μm (Contour, Boston Scientific, Marlborough, MA). The angiographic endpoint for the procedure was identified as a reduction in flow or near stasis in a prostate artery (Figure 3).

In each instance, bilateral prostate artery therapy was tried. After the procedure, there were no complications. The day following the procedure, patients were sent home with non-steroidal anti-inflammatory medications and antibiotics. After that, they were advised to continue taking the antibiotics for another seven days.

Follow-up

All patients’ follow-up results at three months were recorded. Transabdominal ultrasound imaging was performed to assess prostate volume and PVR. Scores on the IPSS and QOL were recorded.

Statistical analysis

The data were verified and evaluated by employing SPSS statistical software (IBM SPSS Statistics for Windows, IBM Corp., Version 25, Armonk, NY) after all of the data had been recorded and input into a Microsoft Excel sheet. The mean as well as standard deviation (SD) had been employed to summarize quantitative variables. Frequency and percentage were employed to express categorical variables. The statistical significance of the difference between the means of the variables before and after the intervention was examined by employing a paired t-test. A p-value < 0.05 had been considered to be statistically significant.

## Results

A total of 25 men underwent PAE treatment, with a mean age of 76.56 ± 7.53 years (between 58 and 91) and an average baseline prostate volume of 86.64 ± 38.69 cm^3^ (ranging from 34 to 178 cm^3^) as measured by ultrasonography. A comprehensive overview of the baseline patient characteristics can be found in Table 1.

**Table 1:** Baseline patient characteristics IPSS, International Prostate Symptom Score; PVR, post-void residual; QoL, Quality of Life

Characteristic	Mean (range)	SD
Age (years)	76.56 (58-91)	7.53
Prostate volume (cm^3^)	86.64 (34-178)	38.69
PVR (cm^3^)	179.12 (40-520)	148.91
IPSS	20.96 (14-25)	3.06
QoL	4.44 (3-5)	0.82

PAE was successfully performed in 23 cases with bilateral embolization, achieving a success rate of 92%. Two unilateral embolizations were conducted due to challenges in identifying the prostate artery. The duration of the PAE procedure ranged from 65 to 210 minutes, with an average time of 111.8 minutes (±41.16). Fluoroscopy time varied between 20 and 145 minutes, averaging 59.56 minutes (±35.51). The average volume of iodinated contrast used was 122.4 mL (±54.6), with a range of 70 to 280 mL. Each procedure utilized between 5 and 28 mL of a 150-250 μm PVA particle solution, averaging 15.16 mL (±4.95).

At three months, follow-up information was gathered for every patient. The means of the baseline and follow-up values were displayed, together with their SDs and ranges. A paired t-test was employed to compare the mean values at various time points. Prostate volume, PVR, IPSS, and QoL scores were measured at the three-month follow-up. All comparisons showed p < 0.001, and the mean maximum improvements were as follows: PVR volume, 70.08 ± 39.85 (52% ± 14.14); prostate volume decrease, 49.40 ± 24.13 (43% ± 13.95); QoL score, 1.80 ± 0.81; and IPSS, 10.44 ± 2.91. A comprehensive summary of BPH characteristics after PAE, along with a comparison to baseline data, can be found in Table 2.

**Table 2:** Benign prostatic hyperplasia characteristics three months after prostatic artery embolization IPSS, International Prostate Symptom Score; PVR, post-void residual; QoL, Quality of Life

Characteristic	Mean ± SD (range)	Percentage change	p-value
Prostate volume (cm^3^)	49.40 ± 24.13 (19-88)	42.73 %	<0.001
PVR (cm^3^)	70.08 ± 39.85 (24-140)	51.89 %	<0.001
IPSS	10.44 ± 2.91 (6-16)	50.99 %	<0.001
QoL	1.80 ± 0.81 (1-3)	60.13 %	<0.001

## Discussion

The effectiveness of PAE as a treatment for BPH is becoming more widely acknowledged. It provides a safe substitute for current surgical procedures such as PUL, TUVP, HoLEP, and TURP. Furthermore, compared to certain surgical techniques, PAE typically preserves erectile and ejaculatory functions better. PAE can improve overall urinary function and alleviate discomfort by shrinking the prostate gland. The procedure is often performed under local anesthesia and conscious sedation, which minimizes the need for general anesthesia and its associated risks, particularly in patients with multiple health issues who may not be suitable for surgery. Patients generally experience a faster recovery with PAE, allowing them to resume normal activities within a few days to a week. Furthermore, PAE can decrease or eliminate the need for long-term medications for BPH, thereby reducing the risk of side effects linked to prolonged medication use. However, a thorough assessment of the potential risks as well as benefits of PAE is essential to determine whether it is the appropriate treatment for an individual's specific condition and circumstances.

It is essential to recognize that anatomical variations in the arterial supply to the prostate can differ among individuals. Interventional radiologists conducting PAE are trained to identify and selectively target the arteries supplying blood to an enlarged prostate, all while minimizing the risk of complications, such as damage to nearby tissues. During the procedure, imaging techniques like angiography are commonly employed to visualize the arterial structure and guide the placement of embolic particles. The success of PAE hinges on accurately targeting the blood vessels supplying the prostate gland and effectively embolizing them to achieve the desired therapeutic results. Outcome metrics for PAE usually include changes in prostate size and urine flow, as well as improvements in symptoms associated with BPH. Prostate volume, PVR, the IPSS, and QOL scores are among the frequent metrics that are compared before the treatment and during follow-up visits to determine both short-term as well as long-term success.

In a prior study, Singhal et al. discovered that after 12 months, patients' IPSS scores had decreased by 30%, and their mean prostate volume had decreased by 22% [8]. Carnevale et al. reported a 91% clinical success rate (including catheter removal and symptom relief) and a 75% technical success rate (defined as bilateral PAE). Following a year, the paired t-test revealed that QoL had greatly improved (mean, 0.4 ± 0.5; p = 0.001), the mean prostate volume decrease had surpassed 30% (p = 0.004), and the mean IPSS score was 2.8 ± 2.1 (p = 0.04) [9]. Our recent study found that after embolization, the mean prostate volume decreased by 43%, the IPSS scores decreased by 51%, and the QoL significantly improved at the three-month follow-up (p < 0.001).

The following mean maximum improvements were observed in the outcomes of PAE for BPH after a three-month follow-up, according to Carnevale et al.: prostatic volume reduction of 39 cm³ ± 39 (39% ± 29); IPSS of 16 points ± 7; QoL score of 4 points ± 1; and PVR volume of 70 mL ± 121 (48% ± 81) (p < 0.05 for all) [10]. Our study aligns with these findings, showing a mean maximum improvement at three months of prostatic volume reduction of 49.40 ± 24.13 (43% ± 13.95); QoL score of 1.80 ± 0.81; IPSS of 10.44 ± 2.91; and PVR volume of 70.08 ± 39.85 (52% ± 14.14) (p < 0.001 for all).

In a single-center retrospective review, Patel et al. demonstrated that PAE for BPH significantly reduced prostate volume and improved symptoms, QoL, and uroflowmetry parameters. Prostate volume decreased from a mean of 156 to 107 mL at 12 months, marking a 27.5% reduction (p < 0.05). Significant improvements were observed in IPSS (21.8 to 10.5) and QoL (4.5 to 2.0), alongside reductions in PVR volume (202 to 105 mL) and increased *Q*_max_ (5.9 to 10.0 mL/second) from baseline to 12 months (p < 0.05). No major complications were reported [11]. 

The retrospective design, short follow-up time, limited sample size, and lack of comparison with surgical procedures are some of the drawbacks of the current study. To better characterize our findings, further prospective, multicenter, and long-term studies are needed.

## Conclusions

For men with BPH, PAE is a successful treatment for symptoms related to the lower urinary tract. For individuals who are unable to undergo surgery because of a variety of comorbidities, it is a good substitute. From an Indian perspective, PAE is a safe way to treat BPH-related LUTS.
